# The association between XPC Lys939Gln gene polymorphism and urinary bladder cancer susceptibility: a systematic review and meta-analysis

**DOI:** 10.1186/1746-1596-8-112

**Published:** 2013-07-02

**Authors:** Kun Dou, Qingzhu Xu, Xiaolu Han

**Affiliations:** 1 Department of Urology, Kunming General Hospital of Chengdu Military Command, Kunming 650032, China; 2 Department of Urology, the Second Hospital of Tianjin Medical University, Tianjin Institute of Urology, Tianjin 300211, China; 3 Department of Urology, The First People’s Hospital of Kunming City, Kunming 650211, China

**Keywords:** Bladder cancer, XPC, Polymorphism, Susceptibility, Meta-analysis

## Abstract

**Background:**

Numerous epidemiological studies have been conducted to explore the association between the Lys939Gln polymorphism of Xeroderma pigmentosum group C (XPC) gene and urinary bladder cancer susceptibility. However, the results remain inconclusive. In order to derive a more precise estimation of this relationship, a large and update meta-analysis was performed in this study.

**Methods:**

A comprehensive search was conducted through researching MEDLINE, EMBASE, PubMed, Web of Science, China Biomedical Literature database (CBM) and China National Knowledge Infrastructure (CNKI) databases before June 2013. Crude odds ratios (ORs) with 95% confidence intervals (CIs) were calculated to estimate the strength of the association.

**Results:**

A total of 12 studies with 4828 cases and 4890 controls for evaluating the XPC Lys939Gln polymorphism and urinary bladder cancer were included. Overall, there was significant associations between the XPC Lys939Gln polymorphism and urinary bladder cancer risk were found for homozygous model (OR = 1.352, 95% CL = 1.088-1.681), heterozygous model (OR = 1.354, 95% CL = 1.085-1.688), and allele comparison (OR = 1.109, 95% CL = 1.013-1.214). In subgroup analysis by ethnicity and source of controls, there were still significant associations detected in some genetic models.

**Conclusion:**

Our meta-analysis suggested that the XPC Lys939Gln polymorphism contributed to the risk of urinary bladder cancer.

**Virtual slides:**

The virtual slide(s) for this article can be found here: .

## Introduction

Bladder cancer is one of the most frequently occurring neoplasms in men. The established risk factors for bladder cancer include cigarette smoking, occupational exposure to certain chemical carcinogens such as aromatic amines and uptake of drugs such as phenacetin and cyclophosphamide. These carcinogens can cause DNA damage, introducing bulky adducts, crosslinks and single or double strand breaks [1]. Tobacco smoke and occupational exposures are the major risk factors in Western countries, whereas schistosoma hematobium infection is the major etiologic factor in developing countries, particularly in Africa and the Middle East [2]. Although many people are exposed to the risk factors, only a fraction of exposed individuals will develop bladder cancer, suggesting an individual susceptibility to the effects of carcinogens.

The xeroderma pigmentosum complementation group C (XPC) protein is involved in the recognition and initiation of the global genome repair pathway of nucleotide excision repair. XPC binds to HR23B to form the stable XPC-HR23B complex, which recognizes and binds to damaged DNA [3]. XPC gene is located at chromosome 3p25 and contains 16 exons and 15 introns, which is one of the eight core genes (XPA, XPB, XPC, XPD, XPE, XPF, XPG and ERCC1) in the NER pathway. Polymorphisms in XPC gene have been evaluated as risk factors in bladder cancer in a number of epidemiological studies, however the conclusions were controversial. Therefore, in the present study, we performed this meta-analysis of all the published studies on bladder cancer to draw a more precise estimation of this association.

## Methods

### Literature search

A comprehensive search was conducted through re.searching MEDLINE, EMBASE, PubMed, Web of Science, China Biomedical Literature database (CBM) and China National Knowledge Infrastructure (CNKI) databases before June 2013. Relevant publications were searched using follow terms: “XPC”, “Xeroderma pigmentosum group C”, “polymorphism”, “variant”, “bladder cancer”, “bladder tumor”, “bladder carcinoma” and “bladder neoplasm”. We evaluated all associated publications to retrieve the most eligible literatures. The reference lists of reviews and retrieved articles were hand searched at the same time. We did not include abstracts or unpublished reports. When overlapping data of the same patient population were included in more than one publication, only the most recent or complete study was used in this meta-analysis.

### Inclusion and exclusion criteria

Studies included in our meta-analysis had to meet the following inclusion criteria: (1) evaluate the association between XPC Lys939Gln polymorphism and bladder cancer risk; (2) case–control designed studies; (3) sufficient information to estimate odds ratios (ORs) and their 95% confidence intervals (CIs). Studies were excluded if one of the following existed: (1) no control population; and (2) duplicate of previous publication.

### Data extraction

Information was carefully assessed and extracted from all eligible publications according to the inclusion criteria by two investigators independently. For conflicting evaluation, an agreement was reached following discussion during a consensus meeting with a third reviewer. The following information was collected from each study: first author's name, year of publication, country of origin, ethnicity, control source (hospital-based or population-based), genotyping methods, and numbers of cases and controls with the XPC different genotypes distribution. The stratification analysis was conducted by ethnicity and control source.

### Statistical methods

Crude odds ratios (ORs) with their corresponding 95% CIs were used to assess the strength of association between the XPC Lys939Gln polymorphism and bladder cancer risk. The pooled ORs and 95% CIs were calculated for Lys939Gln polymorphism using homozygous model (Gln/Gln vs. Lys/Lys), heterozygous model (Lys/Gln vs. Lys/Lys), recessive model [Gln/Gln vs. (Lys/Gln + Lys/Lys)], dominant model [(Lys/Gln + Gln/Gln) vs. Lys/Lys], as well as allele comparison model (Gln vs. Lys). Pooled OR estimate of each study was calculated by both the fixed effects model (the Mantel–Haenszel method) [4] and the random effects model (the DerSimonian and Laird methods) [5]. The fixed-effects model would be adopted when the studies were found to be homogeneous (with P > 0.10 for the Q test). Otherwise, the random-effects model would be applied. Heterogeneity between studies was evaluated by Chi square-based Q-test [6]. Heterogeneity was considered statistically significant if P < 0.10. Heterogeneity was quantified using the *I*^*2*^ metric, which was independent of the number of studies in the meta-analysis (*I*^*2*^ < 25% no heterogeneity, *I*^*2*^ = 25–50% moderate heterogeneity, and *I*^*2*^ > 50% large or extreme heterogeneity). Subgroup analyses were performed by ethnicity and source of control to explore the reasons of heterogeneity. The funnel plot was generated to examine the potential publication bias by using the standard error of log (OR) of each investigation plotted against its log (OR), and the asymmetry of funnel plot was assessed by the method of Egger’s linear regression test [7]. Sensitivity analyses were performed to assess the stability of the results. All statistical tests were performed with Stata (Version 12.0, Stata Corporation, College Station, TX), using two-sided P-values.

## Results

### Study characteristics

A total of 71 potentially relevant publications were identified from the databases listed above. After screening the titles or abstracts, 59 publications were excluded because of duplicate data, case reports, or reviews, or non-relevance research. All studies included in the present meta-analysis used a case–control design. Finally, 12 studies were identified for eligibility and were included in our meta-analysis, with a total of 4,828 cancer cases and 4,890 controls [8-19] (Table 1). All patients in cases groups were histologically or pathologically confirmed, and controls were mainly matched by age, sex and ethnicity. The genotype distribution of the Lys939Gln polymorphism was in compliance with HWE in the controls of all studies.

**Table 1:** Main characteristics of these studies on the associations between XPC Lys939Gln polymorphism and bladder cancer risk

**First author**	**Year**	**Country**	**Ethnicity**	**Study type**	**Genotype method**	**Sample size**		**P**_***HWE***_
						**Case**	**Control**	
Sanyal et al.	2004	Sweden	Caucasian	PB	PCR-RFLP	309	246	0.294
Sak et al.	2005	England	Caucasian	HB	TaqMan	532	561	0.191
Garcia-Closas et al.	2006	Spain	Caucasian	HB	TaqMan	1137	1138	0.470
Wu et al.	2006	USA	Caucasian	HB	TaqMan	606	596	0.744
Zhu et al.	2007	USA	Caucasian	HB	Taqman	550	554	0.668
Fontana et al.	2008	French	Caucasian	HB	TaqMan	51	45	0.456
de Verdier et al.	2010	Sweden	Caucasian	PB	PCR-RFLP	305	328	0.147
Gangwar et al.	2010	India	Asian	HB	PCR-RFLP	208	245	0.054
Rouissi et al.^1^	2011	Tunisia	African	HB	PCR-RFLP	193	193	0.537
Rouissi et al.^2^	2011	Tunisia	African	HB	PCR-RFLP	125	125	0.449
Mittal et al.	2012	India	Asian	PB	PCR-RFLP	212	250	0.073
Liu et al.	2012	China	Asian	HB	PCR-RFLP	600	609	0.824

*PB* Population-Based Study, *HB* Hospital-Based Study, *P*_*HWE*_ P value of Hardy–Weinberg equilibrium. *PCR-RFLP* Polymorphism Chain Reaction- Restriction Fragment Length Polymorphism. *1,2* Different studies.

### Meta-analysis results

**Figure 1:**
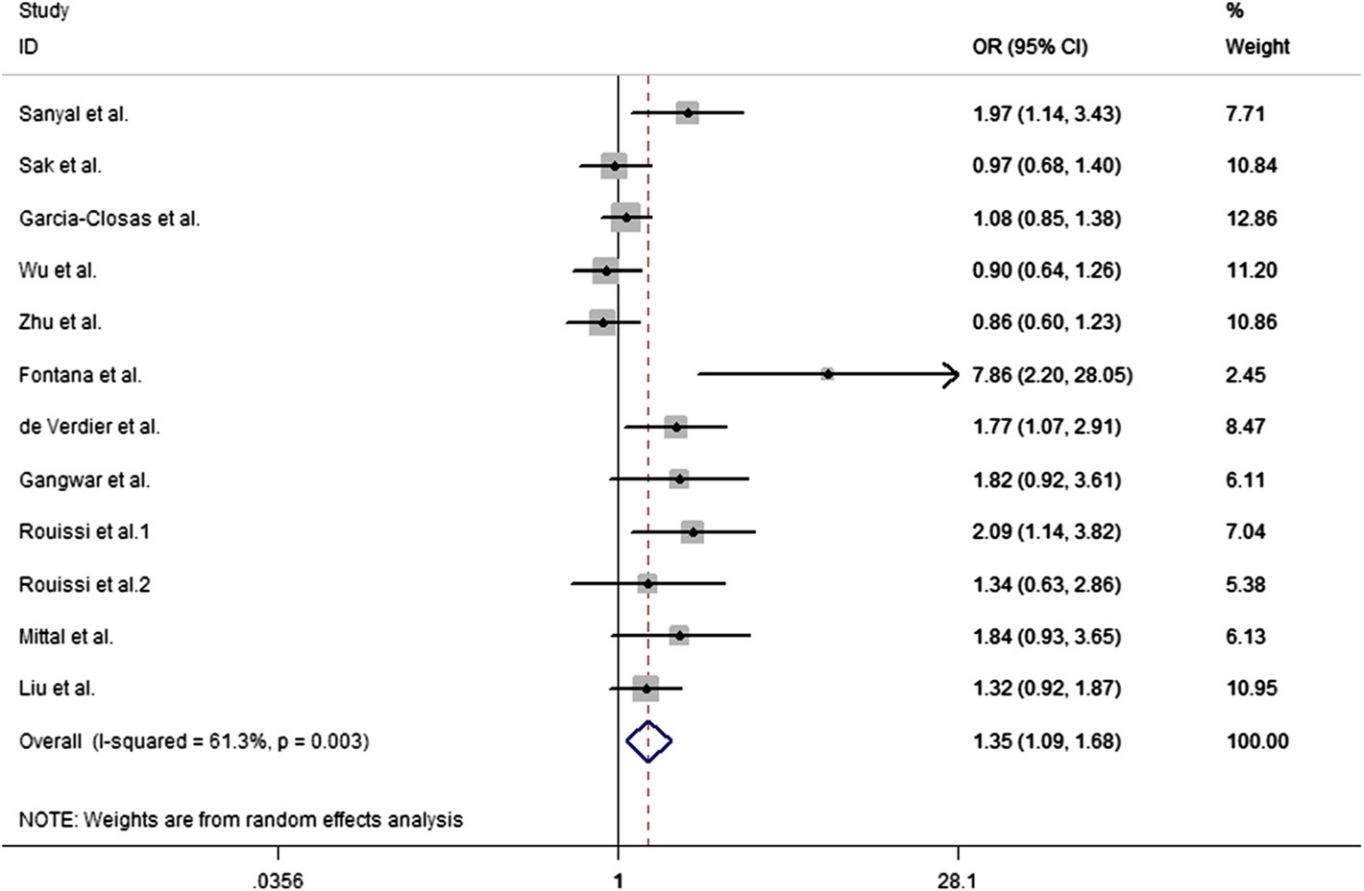
**Forest plots for XPC Lys939Gln polymorphism and risk of bladder cancer in overall populations (homozygous model for Gln/Gln vs Lys/Lys).**

**Figure 2:**
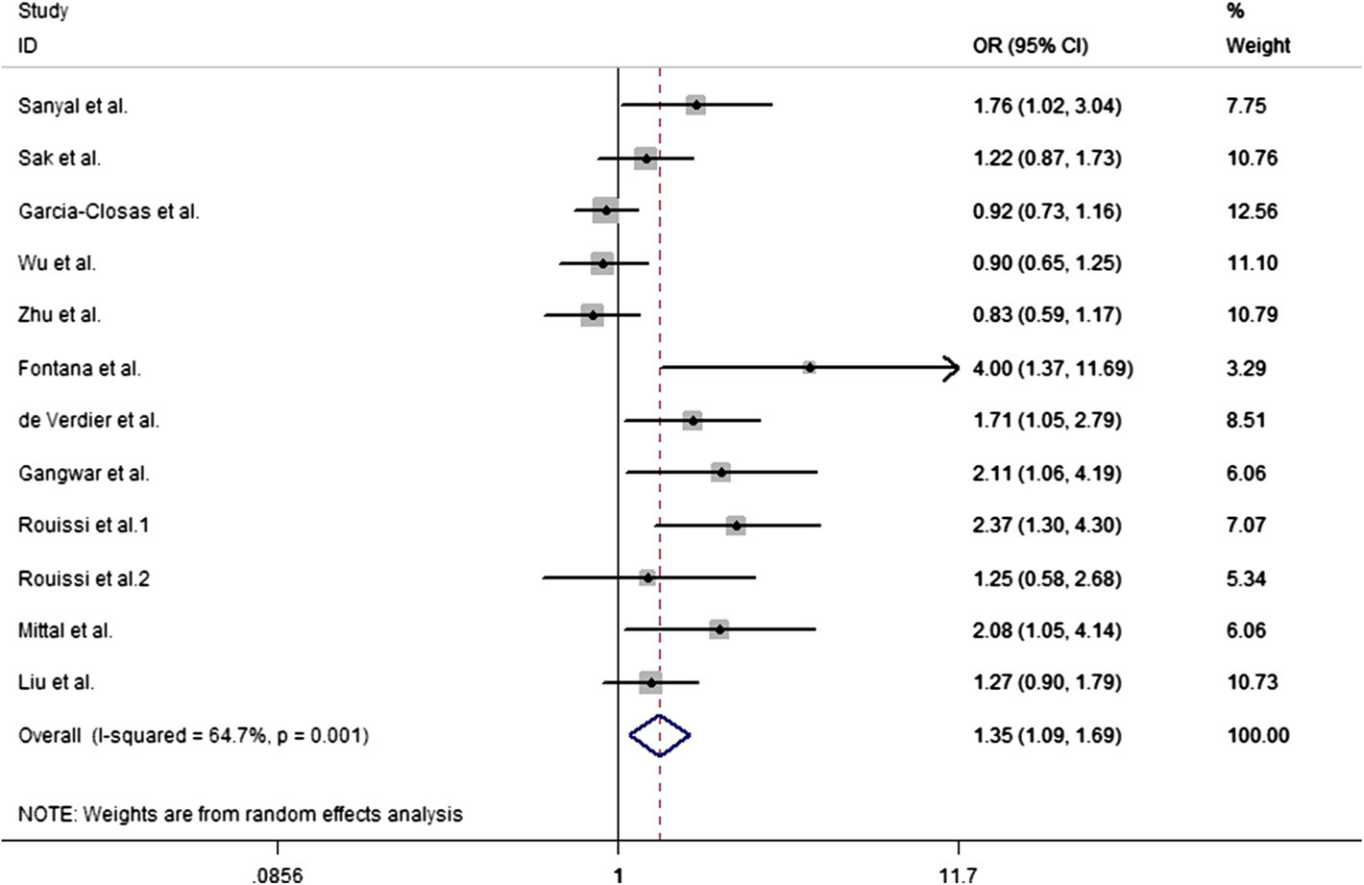
**Forest plots for XPC Lys939Gln polymorphism and risk of bladder cancer in overall populations (heterozygous model for Gln/Gln vs Gln/Lys).**

**Figure 3:**
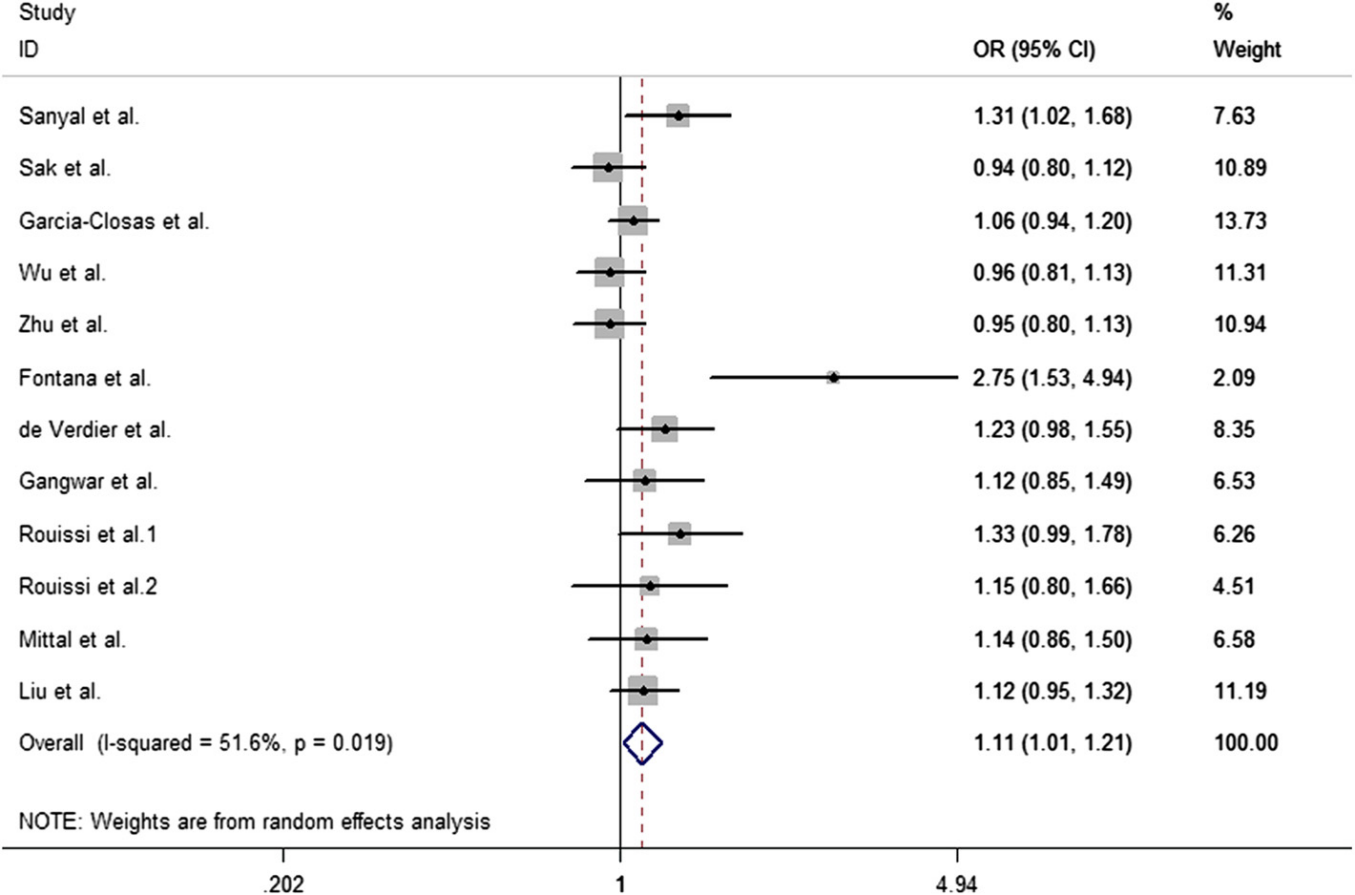
**Forest plots for XPC Lys939Gln polymorphism and risk of bladder cancer in overall populations (allele comparison for Gln allele vs. Lys allele).**

The overall results indicated that there were significant associations between all three studied XPC Lys939Gln polymorphism and increased risk of bladder cancer (Table 2). Overall, there is statistically significant association was found between XPC Lys939Gln polymorphism and the bladder cancer risk in homozygous model (OR = 1.39, 95% CI = 1.08-1.79) (Figure 1), heterozygous model (Gln/Gln vs Gln/Lys: OR = 1.42, 95% CI = 1.11-1.83) (Figure 2), and allele comparison model (OR = 1.12, 95% CI = 1.003-1.24) (Figure 3), but not in other models (Table 2). In the subgroup analysis by ethnicity, statistically significant associations were observed in African populations for homozygous model (OR = 1.758, 95% CI = 1.097-2.819), and recessive model (OR = 1.781, 95% CI = 1.057-3.002). Similarly, statistically significant associations were observed in Asian populations for homozygous model (OR = 1.476, 95% CI = 1.110-1.962), heterozygous model (Gln/Gln vs Gln/Lys: OR = 1.586, 95% CI = 1.105-2.276), and recessive model (OR = 1.489, 95% CI = 1.130-1.962). However, no statistical associations were found in Caucasian populations for other genetic models (Table 2). In population-based populations, statistically significant associations were found for homozygous model (OR = 1.854, 95% CI = 1.338-2.569), heterozygous model (Gln/Gln vs Gln/Lys: OR = 1.806, 95% CI = 1.310 -2.491), recessive model (OR = 1.489, 95% CI = 1.130-1.962) and allele comparison model (OR = 1.233, 95% CI = 1.067-1.423), while there are no statistical associations were found in hospital-based populations for all kinds of comparison models.

**Table 2:** Main results of pooled odds ratios (ORs) with confidence interval (CI) in the meta-analysis

**Variables**	**No. of studies**	**OR (95% CI)**	**Ph**	**P**	**OR (95% CI)**	**Ph**	**P**	**OR (95% CI)**	**Ph**	**P**
		**Gln/Gln vs Lys/Lys**			**Gln/Gln vs Gln/Lys**			**Gln/Lys vs Lys/Lys**		
Total	9	1.352 (1.088 1.681)	0.000	**0.00**	1.354 (1.085 1.688)	0.001	**0.007**	1.017 (0.932 1.109)	0.592	0.712
**Ethnicity**										
Caucasian	3	1.240 (0.927 1.658)	0.002	0.148	1.184 (0.913 1.536)	0.006	0.203	1.041 (0.938 1.155)	0.278	0.448
African	3	1.758 (1.097 2.819)	0.373	**0.019**	1.800 (0.969 3.342)	0.196	0.063	0.955 (0.680 1.341)	0.575	0.791
Asian	2	1.476 (1.110 1.962)	0.554	**0.007**	1.586 (1.105 2.276)	0.256	**0.012**	0.962 (0.802 1.154)	0.655	0.677
**Source of controls**										
HB	5	1.225 (0.967 1.551)	0.009	0.093	1.233 (0.969 1.570)	0.88	0.088	1.017 (0.924 1.120)	0.382	0.728
PB	3	1.854 (1.338 2.569)	0.958	**0.000**	1.806 (1.310 2.491)	0.896	**0.000**	1.013 (0.823 1.247)	0.678	0.900
**Variables**	**No. of studies**	**OR (95% CI)**	**Ph**	**P**	**OR (95% CI)**	**Ph**	**P**	**OR (95% CI)**	**Ph**	**P**
		**Lys/Lys vs Gln/Gln + Gln/Lys (dominant model)**			**Gln/Gln vs Gln/Lys + Lys/Lys (recessive model)**			**Gln allele vs Lys allele**		
Total	9	0.945 (0.867 1.029)	0.397	0.194	1.362 (1.099 1.688)	0.18	0.18	1.109 (1.013 1.214)	0.019	**0.024**
**Ethnicity**										
Caucasian	3	0.940 (0.814 1.085)	0.086	0.397	1.219 (0.934 1.591)	0.002	0.145	1.097 (0.960 1.254)	0.003	0.175
African	3	0.890 (0.649 1.220)	0.948	0.468	1.781 (1.057 3.002)	0.241	**0.030**	1.258 (1.001 1.579)	0.546	0.049
Asian	2	0.954 (0.803 1.133)	0.846	0.590	1.489 (1.130 1.962)	0.357	**0.005**	1.123 (0.989 1.275)	0.996	0.073
**Source of controls**										
HB	5	0.962 (0.865 1.070)	0.264	0.480	1.239 (0.982 1.563)	0.003	0.071	1.078 (0.971 1.197)	0.021	0.160
PB	3	0.874 (0.718 1.065)	0.655	0.183	1.824 (1.343 2.477)	0.956	**0.000**	1.233 (1.067 1.423)	0.749	**0CPA004**

*OR* Odds Ratio, *CL* Confidence Interval, *P*_*h*_ P value of heterogeneity.

### Heterogeneity analyses

Substantial heterogeneities were observed in the overall analysis evaluating the association between XPC Lys939Gln polymorphism and bladder cancer risk in homozygous model (P = 0.000), heterozygous model (Gln/Gln vs Gln/Lys: P = 0.007), and allele comparison (P = 0.007), except for the heterozygous (Gln/Lys vs Lys/Lys: P = 0.348), dominant model (P = 0.397), and recessive model (P = 0.18).

### Sensitivity analyses

We next performed a leave-one-out sensitivity analysis to determine whether a particular study or studies would result in heterogeneity. It was found that one study [13] with small sample size, 51 cases and 45 controls, and obvious genotype frequency deviation may qualitatively change the pooled ORs for Lys939Gln polymorphism. After this study was dropped, the degree of heterogeneity dramatically decreased, without altering the overall estimates.

### Publication bias

**Figure 4:**
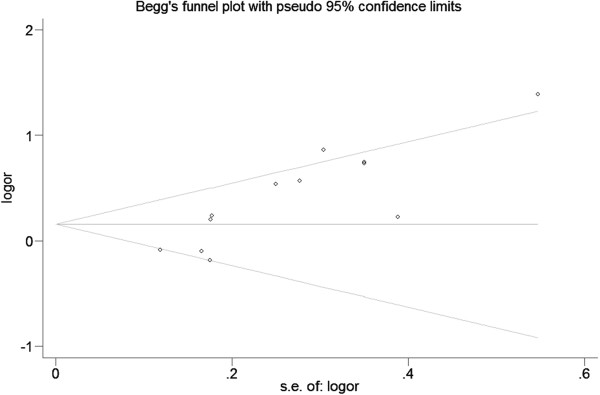
Begg’s funnel plot for assessing the publication bias under the allele contrast model (allele comparison for Gln allele vs. Lys allele).

Begg’s funnel plot and Egger’s test were performed to assess the publication bias. The shape of funnel plots did not reveal any evidence of obvious asymmetry in all comparison models, and the results of Begg’s test did not show any evidence of publication bias (Figure 4).

## Discussion

It is well recognized that there is a range of individual susceptibility to the same kind of cancer even with identical environmental exposure. Host factors, including polymorphisms of genes involved in carcinogenesis, may have accounted for this difference. Therefore, genetic susceptibility to cancer has been a research focus in scientific community.

This meta-analysis, including 4,828 cancer cases and 4,890 controls from 12 published case–control studies, explored the association between the XPC Lys939Gln polymorphism and bladder cancer risk. Overall, we found that there was evidence that the variant genotypes of the XPC Lys939Gln were associated with a significant increased overall risk of bladder cancer. In the subgroup analysis based on ethnicities, significant associations were found between both African and Asian populations for some genetic models, suggesting that XPD Lys939Gln polymorphism play similar roles in populations with different genetic backgrounds and living environment. Simultaneously, our results also showed that significantly increased bladder cancer risk in homozygous, heterozygous, recessive and allele comparison models were noted in the population-based studies but not in hospital-based studies.

There are some limitations should be acknowledged in this meta-analysis. Firstly, bladder cancer is a multi-factorial disease that results from complex interactions between many genetic and environmental factors. It suggests that there will not be single gene or single environmental factor that has large effects on bladder cancer susceptibility. Secondly, in the subgroup analyses by ethnicity and source of controls, the number of subjects was relatively small, not having enough statistical power to explore the real association. Thirdly, the controls were not uniformly defined. Although most of the controls were selected mainly from healthy populations, some had respiratory disease. Therefore, non-differential mis-classification bias was possible because these studies may have included the control groups who had different risks of developing bladder cancer. Fourth, our meta-analysis is similarly with other works[20-22], while a more precise analysis should be conducted if individual data were available, which would allow for the adjustment by other covariates including age, sex, family history, environmental factors and lifestyle. Furthermore, other pathological type of urinary bladder cancer should be considered [23-25], which may be the source of publication. In addition, as in most meta-analyses, publication bias must be considered because only published studies were included in the meta-analysis.

Despite some limitations listed above, our meta-analysis had several advantages. First, sufficient number of cases and controls were pooled from different studies, which significantly increased the statistical power of the analysis. Second, no publication biases were detected, indicating that there is no bias among the pooled results.

## Conclusion

In conclusion, this meta-analysis indicates that XPC Lys939Gln polymorphism may be contributed to the development of bladder cancer risk. However, a study with the larger sample size is needed to further evaluated gene-environment interaction on XPC Lys939Gln polymorphism and bladder cancer risk.

## Competing interests

None of the authors have any conflict of interests to declare.

## Authors’ contributions

KD and QX carried out the meta-analysis study, drafted the manuscript and involved in revising the manuscript critically for important intellectual content. XH and QX participated in the design of the study and revised the manuscript. KD and QX carried out the meta-analysis study and drafted the manuscript. KD participated in the design of the study, drafted the manuscript and revised the manuscript. All authors read and approved the final manuscript.
